# Comparison of a Proposed Strength Training Program Using Indirect 1RM Versus Mobile Application Recommendations for Body Fat Loss: A Quasi-Experimental Study

**DOI:** 10.3390/sports14050169

**Published:** 2026-04-22

**Authors:** Julio Alberto Morales Viscaya, Ricardo López Garcia, José Omar Lagunes Carrasco, Erik Ramirez López, Ximena Martínez Mireles

**Affiliations:** Facultad de Organizacion Deportiva, Universidad Autónoma de Nuevo León, Torre de Rectoría Ubicada en Pedro de Alba S/N, Entre Av. Alfonso Reyes y Av. Fidel Velázquez, San Nicolás de los Garza C.P. 66455, Mexico; ricardo.lopezgr@uanl.edu.mx (R.L.G.); erik.ramirezlp@uanl.edu.mx (E.R.L.); ximenamtznutricion@gmail.com (X.M.M.)

**Keywords:** obesity, strength training, indirect 1RM, mobile application, body composition, supervised exercise

## Abstract

Obesity represents a major public health crisis in Mexico, affecting over 70% of adults, and although strength training is effective for improving body composition, direct maximal strength testing (1RM) poses risks in this population. Mobile applications have emerged as popular tools for exercise prescription, yet their effectiveness compared to supervised, scientifically-based protocols remains unknown. To compare the effects of a supervised strength training program based on indirect 1RM estimation (S1RM group) versus a mobile application-generated program (App group) on body composition, anthropometric measures, and strength gains in male adults with obesity. Twenty male participants (BMI ≥ 30 kg/m^2^) were randomly assigned to either the S1RM group or the App group. Both groups trained three times per week for 12 weeks. Body composition (bioelectrical impedance), anthropometric measures (waist and hip circumference), and estimated 1RM were assessed pre- and post-intervention. A mixed repeated-measures ANOVA (Group × Time) was conducted, with effect sizes ($\eta^{2}$p) and 95% confidence intervals calculated. Both groups showed significant improvements in most outcomes (*p* < 0.05). However, significant group × time interactions favored the S1RM group for waist circumference (F(1,18) = 14.50, *p* = 0.001, $\eta^{2}$*p* = 0.45) and hip circumference (F(1,18) = 217.90, *p* < 0.001, $\eta^{2}$*p* = 0.92). A significant between-group difference was also observed for visceral fat (F(1,18) = 4.91, *p* = 0.040, $\eta^{2}$*p* = 0.21). For muscle and fat mass, interactions showed large effect sizes ($\eta^{2}$*p* = 0.18–0.19) with trends toward significance (*p* = 0.057–0.096). Strength increased significantly in all exercises for the S1RM group (14.9–22.0%, *p* < 0.01). These findings support the implementation of indirect 1RM estimation methods in obesity populations and highlight the added value of professional supervision in strength training programs.

## 1. Introduction

Obesity and overweight represent one of the most pressing public health crises in Mexico. Recent data indicate that more than seven out of ten Mexican adults carry excess weight, with a particularly alarming prevalence of 83.6% among individuals in their fourth decade of life. Between 2000 and 2018, obesity rates in the country increased by 42.2%, with a disproportionate rise in its most severe forms: severe obesity surged by 70.1%, while morbid obesity increased by 96.5% [1]. This worrying trend among adults is mirrored in younger populations; data from the National Health and Nutrition Continuous Survey (ENSANUT) 2020–2024 reveal that the combined prevalence of overweight and obesity remains critically high, affecting 36.6% of school-aged children and 40.1% of adolescents [2]. These figures reflect not only a metabolic and nutritional problem but also a significant challenge for healthcare and physical activity systems, which must adapt their strategies to the specific needs and limitations of this population [3]. The importance of addressing this issue is underscored by global health authorities; the World Health Organization emphasizes that regular physical activity is a cornerstone for the prevention and management of non-communicable diseases, including those related to excess body weight [4]. Furthermore, the benefits of an active lifestyle are amplified when combined with balanced dietary patterns, which together form a comprehensive strategy for long-term health maintenance [5,6]. Indeed, the positive effects of physical activity extend beyond weight control, contributing to a reduction in systemic inflammation and the risk of developing chronic diseases such as type 2 diabetes and coronary heart disease [7,8].

In this context, strength training emerges as a fundamental tool for improving body composition and metabolic health. However, its safe and effective prescription for individuals with obesity is complicated by the risks associated with the direct assessment of maximal strength (1RM) [9]. Implementing direct 1RM testing in this population carries a high risk of musculoskeletal injuries and adverse cardiovascular events due to the extreme loads, potential lack of proper technique, joint limitations, and the hemodynamic stress induced by a maximal effort. To overcome these limitations, indirect methods for strength assessment present a viable and safer alternative. These approaches, widely validated in athletic populations to estimate one-repetition maximum (1RM) without requiring a true maximal effort, can be strategically applied to individuals with obesity. In particular, the Brzycki equation [10] offers a practical and accessible formula for translating submaximal performance into an estimated 1RM value, enabling practitioners to prescribe individualized training loads while mitigating the inherent risks of direct testing [11].

This study contributes to the field by introducing a structured, accessible, and clinically applicable model for strength training in populations with obesity. Its primary innovations are threefold. First, it adapts and validates the use of the Brzycki equation—a well-established indirect method for one-repetition maximum (1RM) estimation—specifically for individuals with a high body mass index, offering a safer alternative to direct testing. Second, it proposes a standardized exercise selection protocol consisting of accessible, machine-based exercises (e.g., chest press, leg extension, seated row) that comprehensively target all major muscle groups, ensuring practicality and scalability in common gym settings. Third, the research design incorporates a comparative analysis between two active intervention arms: the Supervised 1RM Estimation Group, which follows the proposed indirect method-based program under professional supervision, and the Mobile Application Group, which adheres to training recommendations generated by a popular fitness app (Smart Fit App). This direct statistical comparison of pre- and post-intervention variations in body composition, strength gains, and adherence rates between the two groups provides empirical evidence on the relative effectiveness of supervised, scientifically planned training versus digital, algorithm-driven exercise prescription in managing obesity.

## 2. Materials and Methods

### 2.1. Study Design and Participants

A quasi-experimental study was conducted with two active intervention arms. The study population consisted of 20 male participants residing in the municipality of San Pedro Garza García, Nuevo León, Mexico. Participants were recruited through convenience sampling and randomly assigned to one of two groups: the Supervised 1RM Estimation Group (S1RM Group, n = 10) or the Mobile Application Group (App Group, n = 10). The sample size was determined by the number of participants who met the inclusion criteria and completed the intervention during the recruitment period.

Inclusion criteria were: (1) voluntary agreement to participate and provision of written informed consent; (2) body mass index (BMI) ≥30 kg/m^2^; (3) being a beginner in structured physical training (less than 3 months of previous experience); (4) availability to attend at least three supervised training sessions per week; and (5) availability of at least 45 min per training session.

Exclusion criteria were: (1) any condition preventing the completion of bioelectrical impedance analysis; (2) undergoing specific medical treatment for obesity (e.g., pharmacotherapy or awaiting bariatric surgery); or (3) presenting any clinical condition contraindicated for moderate-to-vigorous strength training (e.g., uncontrolled cardiovascular, musculoskeletal, or metabolic disorders). An elimination criterion was established for participants who did not complete at least 80% of the total scheduled training sessions.

#### Ethical Considerations and Informed Consent

All participants received comprehensive information regarding the study objectives, procedures to be followed, potential benefits, expected risks or discomforts, and their rights as participants, including the right to withdraw from the study at any time without penalty. This process was conducted in accordance with Articles 20 and 21 of the General Health Law on Health Research [12]. Written informed consent was obtained from each participant prior to the initiation of any study-related procedures. The confidentiality and privacy of all personal data were rigorously protected; data were anonymized and depersonalized before analysis and were accessible only to the research team for the exclusive purposes of the study.

Ethical approval for the present study was obtained retrospectively after the intervention had concluded, as the study was initially developed within the context of an academic program rather than as a prospectively designed trial. The study consisted of a retrospective analysis of the effects of a supervised exercise program on body composition, assessed using bioelectrical impedance analysis (InBody), employing non-invasive procedures that are routinely used in training environments and entail no more than minimal risk to participants. In accordance with Article 17 of the Regulations of the General Health Law on Health Research, the study is classified as research involving no risk, as it is based on previously generated data and non-invasive procedures. The study protocol was approved by the Institutional Ethics Committee of the Instituto Tecnológico de Sonora (Approval No. 564, dated 23 March 2026).

### 2.2. Assessment of Outcome Measures

All anthropometric and body composition measurements were performed in the exercise physiology laboratory under controlled environmental conditions (temperature: 21–22 °C, natural ventilation, neutral and cool lighting). A bioelectrical impedance scale (InBody 270, InBody Co., Ltd., Seoul, Republic of Korea) was used to assess body weight, muscle mass, fat mass, visceral fat percentage, and BMI. Waist and hip circumferences were measured using a non-elastic anthropometric tape (Seca 201, Seca GmbH & Co., Hamburg, Germany), following standardized protocols from the World Health Organization. All measurements were performed by a certified anthropometrist (ISAK Level 2), under fasting conditions of at least 4 h and without having performed physical exercise in the previous 24 h.

### 2.3. Training Protocol for the S1RM Group

Participants in the S1RM Group followed a structured training program based on indirect estimation of maximal strength (1RM) using the Brzycki equation. The training protocol consisted of 45 min sessions, conducted three times per week for 12 weeks. Training intensity was progressively increased from 60% to 75% of the estimated 1RM over the intervention period. Each session included 8 to 12 repetitions per set across 8 to 10 exercises, incorporating both multi-joint (compound) and single-joint (isolation) movements targeting all major muscle groups. The repetition cadence was performed with a 3-1-2-0 tempo: 3 s for the eccentric phase, 1 s pause at the stretched position, 2 s for the concentric phase, and no pause at the contracted position. The exercises evaluated and subsequently included in the training program were: chest press, lat pulldown, shoulder press, triceps extension, biceps curl, leg extension, and seated leg curl.

**Figure 1:**
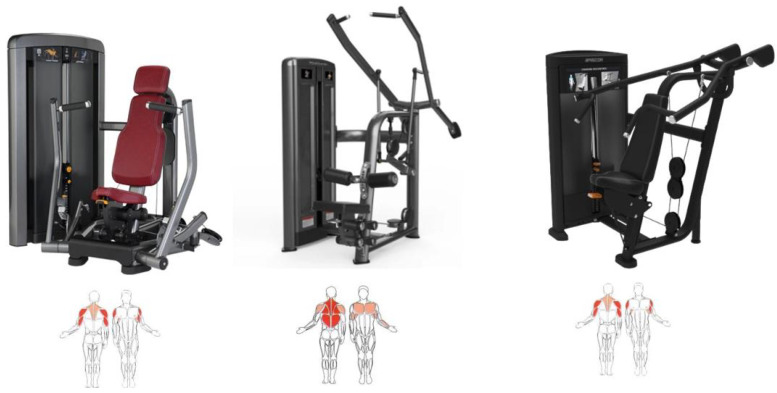
Chest-press, pull-down and shoulder-press machines and the muscles that are exercised. Below, a human body is shown with the exercised muscles highlighted in red.

**Figure 2:**
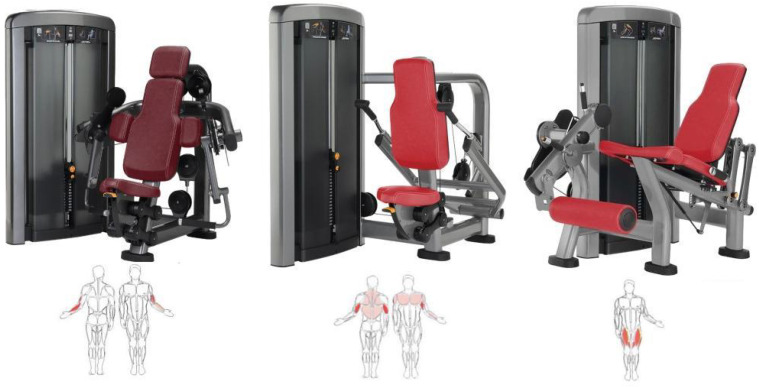
Biceps-curl, triceps-press and leg-extension machines and the muscles that are exercised. Below, a human body is shown with the exercised muscles highlighted in red.

**Figure 3:**
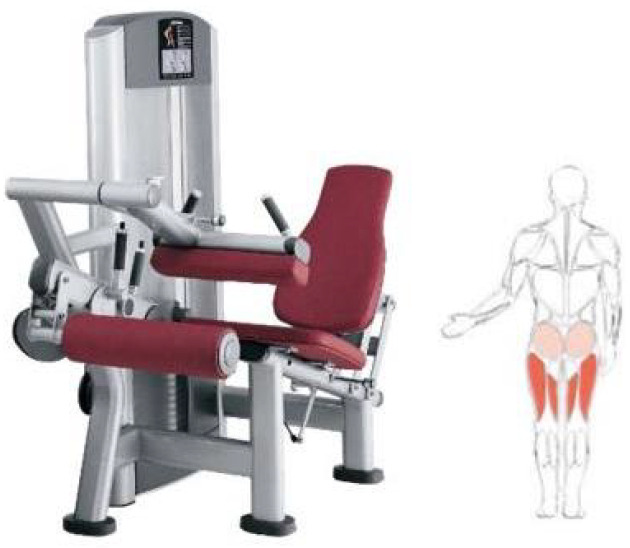
Seated leg curl machine for the last proposed exercise and the muscles that are exercised.

In Table 1 you can see the exercises included in the training program with their primary and synergistic muscle targets. Figure 1, Figure 2 and Figure 3 show the equipment needed to perform the proposed exercises.

### 2.4. Indirect Strength Assessment Protocol: The Brzycki Equation Method

To safely and accurately estimate the one-repetition maximum (1RM) for training prescription, an indirect assessment protocol was implemented using the Brzycki equation [10]. Prior to the 1RM estimation, participants performed a standardized warm-up consisting of 5 min of light cardio and dynamic stretching. For each of the seven key exercises (chest press, lat pulldown, shoulder press, triceps extension, biceps curl, leg extension, and seated leg curl), participants first performed a set of 10 repetitions with a light, self-selected load to familiarize themselves with the movement. Following this, they performed a single submaximal set to volitional failure with a load they could lift for approximately 8–12 repetitions. A certified trainer closely supervised each attempt to ensure proper form and full range of motion. The load lifted (in kg) and the exact number of repetitions completed to muscular failure were recorded. The Brzycki equation was then applied to calculate the estimated 1RM for each exercise:

(1) $$1RM_{est}=\frac{Load}{\left(1.0278-(0.0278\times Repetitions)\right)}$$

where *Load* is the weight used in the submaximal set (kg) and *Repetitions* is the number of repetitions performed to failure. This estimated 1RM value served as the baseline reference for prescribing training intensities throughout the 12-week intervention. Training loads for the S1RM Group were calculated as percentages (60–75%) of this estimated 1RM, ensuring progressive and individualized overload while eliminating the risks associated with maximal direct testing.

### 2.5. Mobile Application Intervention Protocol: Smart Fit App

The App Group followed a training program generated by the Smart Fit App, (version not specified by developer, accessed 4 March 2026 via Google Play) a commercially available fitness application. This application was selected as a comparator due to its popularity, accessibility, and representative nature of widely used digital fitness tools, allowing for a pragmatic comparison between a supervised, scientifically prescribed training protocol and a common real-world approach based on algorithmic, self-directed exercise prescription.

The application is available on both iOS and Android operating systems, with high user ratings (iOS: 4.8/5 based on over 14,000 ratings; Android: 4.9/5 based on over 198,000 ratings). It is ranked #12 among free applications in the Health and Fitness category and has been downloaded more than 5 million times.

Upon initial setup, the application prompts users to input personal and fitness-related information to generate a tailored workout plan. The parameters collected include height and current body weight, previous experience in gym training (beginner, intermediate, advanced), training routine during the previous four months (active or inactive), and desired training frequency (number of sessions per week). Based on these inputs, the application’s algorithm designs a weekly exercise plan consisting of machine-based and free-weight exercises similar to those used in the S1RM Group. The recommended training volume typically ranged from 8 to 12 repetitions per exercise, though the load was self-selected by the participant based on perceived exertion, without reference to an estimated or tested 1RM.

Participants in the App Group were instructed to follow the application’s recommendations independently, without in-person supervision. They were provided with access to the application and a brief orientation on its basic functions but received no further guidance on exercise technique, progression, or load adjustment. Training frequency was set to three sessions per week to match the S1RM Group, with each session lasting approximately 45 min. Adherence was monitored through self-reported training logs and periodic verification of the application’s internal tracking data. It is acknowledged that this monitoring method constitutes a limitation, as it may introduce social desirability bias or inaccuracies in recording.

The APP was selected as a comparator due to its popularity, accessibility, and representative nature of widely used digital fitness tools. This allowed for a pragmatic comparison between a supervised, scientifically prescribed training protocol and a common real-world approach based on algorithmic, self-directed exercise prescription.

### 2.6. Statistical Analysis

Statistical analysis will be performed using jamovi (Version 2.6) [13], a free and open statistical platform. Given the exploratory nature of the study and the sample size determined by participant availability (n = 10 per group), the following approach will be used.

Normality of data distribution will be verified using the Shapiro-Wilk test, and homogeneity of variances will be assessed using Levene’s test. For variables meeting normality assumptions, parametric tests will be used; otherwise, non-parametric equivalents will be employed.

Baseline characteristics between groups will be compared using independent samples *t*-tests or Mann-Whitney U tests, as appropriate. To evaluate the intervention effects, a mixed repeated-measures analysis of variance (two-way ANOVA: Group × Time) will be conducted, with group as the between-subjects factor (S1RM vs. App) and time as the within-subjects factor (pre- vs. post-intervention). Effect sizes will be calculated using partial eta squared ($\eta^{2}p$) for main effects and interactions, interpreted as small ($\eta^{2}p\geq 0.01$), medium ($\eta^{2}p\geq 0.06$), and large ($\eta^{2}p\geq 0.14$). Additionally, 95% confidence intervals for pre–post differences within each group and for between-group differences will be calculated. Greenhouse–Geisser correction will be applied when the sphericity assumption is violated. Statistical significance will be set at α=0.05, though given the small sample size, *p*-values will be interpreted with caution alongside effect sizes and confidence intervals to assess practical significance.

## 3. Results

### 3.1. Baseline Characteristics and Between-Group Comparisons

No significant differences were observed between the S1RM and App groups at baseline for any of the outcome variables (all p>0.05), confirming that the randomization procedure successfully established comparable groups. Normality was assessed using the Shapiro–Wilk test, revealing that all variables except visceral fat met the assumption of normality (p>0.05). For visceral fat, non-parametric tests were used accordingly. Table 2 presents the baseline characteristics and between-group comparisons.

### 3.2. Changes in Body Composition and Anthropometric Measures

Table 3 presents the pre- and post-intervention values for both groups, along with the mean differences (Δ). Both groups showed improvements in most variables, with the S1RM group exhibiting larger reductions in weight, BMI, fat mass, and waist and hip circumferences.

A mixed repeated-measures ANOVA (Group × Time) was conducted to evaluate the statistical significance of these changes. Table 4 summarizes the F-values, *p*-values, and partial eta squared ($\eta^{2}p$) effect sizes for the main effects of Time and Group, as well as their interaction.

A significant main effect of time was observed for all variables except fat mass percentage (p=0.182), indicating that both interventions produced significant improvements in most outcomes. Critically, significant group × time interactions were found for waist circumference ($F_{\left(1,18\right)}=14.50$, p=0.001, $\eta_{p}^{2}=0.45$) and hip circumference ($F_{\left(1,18\right)}=217.90$, p<0.001, $\eta_{p}^{2}=0.92$), demonstrating that the S1RM group experienced greater reductions in these measures compared to the App group. A significant between-group difference was also observed for visceral fat ($F_{\left(1,18\right)}=4.91$, p=0.040, $\eta_{p}^{2}=0.21$), with the S1RM group showing lower overall values. For muscle mass and fat mass, the group × time interactions showed clear trends (p=0.057, 0.080, 0.093, and 0.096) with large effect sizes ($\eta_{p}^{2}$ ranging from 0.18 to 0.19), but did not reach conventional statistical significance, likely due to the limited sample size.

### 3.3. Pre–Post Differences and Confidence Intervals

To complement the ANOVA results and provide a more comprehensive assessment of the intervention effects, Table 5 presents the mean pre–post differences (Δ) along with their 95% confidence intervals (CI) for both groups. The confidence intervals offer insight into the precision and clinical significance of the observed changes, which is particularly relevant given the small sample size.

Notably, the confidence intervals for waist circumference reduction in the S1RM group ranged from −9.82 cm to −3.18 cm, indicating a clinically meaningful reduction that did not overlap with the App group’s interval (−1.52 cm to −0.48 cm). This non-overlap provides additional evidence for the superiority of the S1RM protocol in reducing central adiposity. For muscle mass and fat mass variables, the confidence intervals were wider in the S1RM group, reflecting greater inter-individual variability in response to the supervised protocol.

### 3.4. Strength Gains in the S1RM Group

Table 6 presents the changes in estimated maximal strength (indirect 1RM) for the S1RM group. Paired *t*-tests revealed statistically significant increases for all seven exercises (p<0.01), with improvements ranging from 14.9% to 22.0%.

### 3.5. Adherence and Attendance

The average attendance rate was 91.72% (SD = 4.44%) in the S1RM group and 88.64% (SD = 5.72%) in the App group, indicating good compliance with the prescribed training frequency in both groups. No adverse events were reported in either group during the 12-week intervention period.

## 4. Discussion

The present study compared the effects of a supervised strength training program based on indirect 1RM estimation (S1RM group) versus a mobile application-generated program (App group) on body composition, anthropometric measures, and strength gains in male adults with obesity. The main findings reveal that both interventions produced significant improvements in most outcome measures. However, the S1RM protocol demonstrated statistically superior effects for waist circumference, hip circumference, and visceral fat, with large effect sizes. For muscle and fat mass, the S1RM group showed trends toward greater improvements with large effect sizes, though these did not reach statistical significance, likely due to the limited sample size.

### 4.1. Both Interventions Were Effective

Consistent with previous research on strength training in populations with obesity [14,15], both groups showed significant improvements in body weight, BMI, muscle mass percentage, waist circumference, and hip circumference from pre- to post-intervention (all p<0.001). These findings support the notion that structured strength training—whether supervised and scientifically prescribed or algorithm-generated and self-directed—can induce beneficial changes in body composition among beginners with obesity. This is clinically relevant, as it suggests that multiple accessible options exist for individuals seeking to initiate a training program.

The App group’s improvements are particularly noteworthy given the absence of in-person supervision and the reliance on self-selected loads. These results align with previous research on digital fitness tools [16] and suggest that mobile applications may serve as an accessible alternative for individuals who cannot access supervised training due to economic, geographic, or time constraints.

### 4.2. Advantages of the S1RM Protocol for Central Adiposity

The most striking findings of this study are the significant group × time interactions favoring the S1RM group for waist circumference ($F_{\left(1,18\right)}=14.50$, p=0.001, $\eta_{p}^{2}=0.45$) and hip circumference ($F_{\left(1,18\right)}=217.90$, p<0.001, $\eta_{p}^{2}=0.92$). These effect sizes are considered large, indicating clinically meaningful differences. The S1RM group reduced waist circumference by 6.5 cm (95% CI: −9.82 to −3.18 cm), compared to only 1.0 cm (95% CI: −1.52 to −0.48 cm) in the App group. The non-overlap of confidence intervals provides additional evidence for the superiority of the S1RM protocol.

These findings are particularly important given that central adiposity is strongly associated with cardiometabolic risk, including type 2 diabetes, hypertension, and cardiovascular disease [8,17]. The superior reduction in abdominal and gluteal circumference in the S1RM group may be attributed to the individualized load progression based on estimated 1RM, which ensured a more consistent and progressive overload compared to the self-selected loads in the App group.

Additionally, a significant between-group difference was observed for visceral fat ($F_{\left(1,18\right)}=4.91$, p=0.040, $\eta_{p}^{2}=0.21$), with the S1RM group showing lower overall values. Visceral fat reduction is a critical target in obesity management due to its link to metabolic syndrome and systemic inflammation [11].

### 4.3. Trends in Muscle and Fat Mass

For muscle mass and fat mass, the group × time interactions showed clear trends (p=0.057, 0.080, 0.093, and 0.096) with large effect sizes ($\eta_{p}^{2}$ ranging from 0.18 to 0.19), but did not reach the conventional threshold for statistical significance (p<0.05). The small sample size (n = 10 per group) limited the statistical power to detect moderate differences. The large effect sizes suggest that these differences may be clinically meaningful, and future studies with larger samples may confirm their statistical significance.

The S1RM group gained 1.4 kg of muscle mass (95% CI: +0.52 to +2.28 kg) compared to 1.2 kg (95% CI: +0.45 to +1.95 kg) in the App group, and reduced fat mass by 3.4 kg (95% CI: −5.62 to −1.18 kg) compared to 0.6 kg (95% CI: −2.18 to +0.98 kg) in the App group. The confidence interval for fat mass reduction in the App group included zero, indicating that the change was not statistically significant within that group.

### 4.4. Strength Gains in the S1RM Group

The S1RM group demonstrated significant increases in estimated 1RM for all seven exercises, with improvements ranging from 14.9% to 22.0% (all p<0.01). The largest improvements were observed in leg extension (22.0%) and shoulder press (20.0%), suggesting that lower body and shoulder girdle muscles may respond particularly well to the progressive overload protocol.

### 4.5. Adherence and Safety

The high attendance rates in both groups (S1RM: 91.72%; App: 88.64%) indicate good compliance with the prescribed training frequency. No adverse events were reported in either group, supporting the safety of both interventions. Notably, the indirect 1RM estimation method proved safe, with no musculoskeletal injuries or cardiovascular events during the submaximal testing or the subsequent training sessions. This addresses a key concern raised in the Introduction regarding the risks of direct maximal testing in obesity populations [9].

### 4.6. Limitations

Several limitations must be acknowledged. First, the sample size was small (n = 10 per group) and determined by participant availability rather than an *a priori* power calculation. This limited our ability to detect small-to-moderate differences and may explain why some interactions with large effect sizes did not reach statistical significance.

Second, the study included only male participants, limiting generalizability to women. Future studies should include female participants to assess whether the observed effects are consistent across sexes.

Third, this study did not monitor or control participants’ dietary intake throughout the intervention period. Nutritional habits are a well-established determinant of body composition changes, particularly for fat mass reduction [18]. The absence of dietary control means that some of the observed changes—especially in weight and fat mass—may be partially attributable to variations in energy intake rather than the exercise interventions alone. Future studies should include dietary assessments (e.g., food frequency questionnaires or dietary recalls) to isolate the independent effects of the training protocols.

Fourth, the S1RM protocol combined multiple components: (a) the indirect 1RM estimation using the Brzycki equation, (b) individualized load prescription based on a percentage of estimated 1RM, (c) progressive overload, and (d) professional supervision. Therefore, the specific contribution of the indirect 1RM method itself cannot be isolated from the overall intervention. It is possible that the observed benefits of the S1RM group were due to professional supervision, the structured progression, or the combination of these factors, rather than the estimation method per se.

Fifth, this study did not assess psychological or behavioral variables such as motivation, self-efficacy, or exercise enjoyment, which may influence adherence and long-term outcomes.

### 4.7. Practical Implications

Despite these limitations, our findings have practical implications for exercise professionals working with obesity populations. The S1RM protocol offers a safe, effective, and individualized approach for strength training in obesity, particularly for reducing central adiposity. The significant interactions for waist and hip circumference—two clinically relevant measures of cardiometabolic risk—support the value of supervised, scientifically prescribed training over algorithm-generated programs for these specific outcomes.

For practitioners, the indirect 1RM method using the Brzycki equation provides a practical alternative to direct maximal testing, eliminating injury risk while enabling individualized load prescription. This is particularly relevant in clinical and gym settings where resources for direct testing may be limited.

For individuals who cannot access supervised training, mobile applications may serve as an acceptable alternative, as they also produced significant improvements in most outcomes. However, the superior effects of the S1RM protocol on central adiposity suggest that professional supervision and individualized load prescription provide added value that should be considered when feasible.

## 5. Conclusions

This study compared a supervised strength training program based on indirect 1RM estimation (S1RM group) versus a mobile application-generated program (App group) in male adults with obesity. The main conclusions are as follows:

1. Both interventions were effective: The S1RM and App groups showed significant improvements in body weight, BMI, muscle mass percentage, waist circumference, and hip circumference after 12 weeks of training, demonstrating that structured strength training—whether supervised or app-based—can improve body composition in obesity populations.
2. The S1RM protocol was superior for central adiposity: Significant group × time interactions favored the S1RM group for waist circumference (*p* = 0.001, $\eta^{2}$*p* = 0.45) and hip circumference (*p* < 0.001, $\eta^{2}$*p* = 0.92), with large effect sizes and non-overlapping confidence intervals. A significant between-group difference was also observed for visceral fat (*p* = 0.040, $\eta^{2}$*p* = 0.21).
3. Trends favored the S1RM protocol for muscle and fat mass: Although not statistically significant, the S1RM group showed trends toward greater improvements in muscle mass (*p* = 0.057, $\eta^{2}$*p* = 0.19) and fat mass (*p* = 0.080, $\eta^{2}$ = 0.19; *p* = 0.096, $\eta^{2}$ = 0.18) with large effect sizes, suggesting clinical relevance that may be confirmed in larger studies.
4. Strength gains were significant in the S1RM group: Estimated 1RM increased by 14.9–22.0% in all seven exercises (*p* < 0.01), demonstrating the effectiveness of the indirect method for progressive overload prescription.
5. The indirect 1RM method was safe: No adverse events were reported, confirming that the Brzycki equation provides a safe alternative to direct maximal testing in obesity populations.

In conclusion, the supervised S1RM protocol demonstrated superior effects on central adiposity (waist and hip circumference) and visceral fat compared to the mobile application-based program, with large effect sizes and clinically meaningful differences. Both interventions improved overall body composition, suggesting that mobile applications may serve as an acceptable alternative when supervision is not feasible. However, the added benefits of professional supervision and individualized load prescription—particularly for reducing cardiometabolic risk—support the implementation of indirect 1RM estimation methods in obesity populations. These findings contribute to the growing evidence base for safe and effective strength training in individuals with obesity and provide practical guidance for exercise professionals seeking to optimize training prescription in this population.

## Figures and Tables

**Table 1:** Exercises included in the training program with their primary and synergistic muscle targets.

Exercise	Primary Musculature	Synergists/Stabilizers
Chest Press	Deltoid (clavicular and acromial parts), triceps brachii.	Trapezius (descending part), serratus anterior; pectoralis major (depending on the backrest inclination angle).
Pull Down (Lat Pulldown)	Latissimus dorsi, trapezius, rhomboids, teres major.	Deltoid (acromial part); pectoralis major; biceps brachii; serratus anterior; arm flexor muscles.
Shoulder Press (Overhead Press)	Deltoid (clavicular and acromial parts), triceps brachii.	Trapezius (descending part); serratus anterior; pectoralis major (depending on the backrest inclination angle).
Triceps Press (Triceps Extension)	Triceps brachii.	
Biceps Curl	Biceps brachii, brachialis, brachioradialis.	Forearm flexors.
Leg Extension	Quadriceps femoris; rectus femoris, vastus medialis and lateralis.	
Seated Leg Curl (Hamstring Curl)	Biceps femoris, semitendinosus, semimembranosus.	Sartorius, gracilis, gluteus maximus.

**Table 2:** Baseline characteristics and between-group comparisons (n = 10 per group).

Variable	S1RM Group		App Group		*p*-Value	Cohen’s d
	Mean	SD	Mean	SD		
*Body composition*						
Weight (kg)	98.4	4.5	97.6	7.7	0.193	0.61
BMI (kg/m^2^)	31.6	0.8	32.1	1.6	0.390	0.39
*Muscle mass*						
Muscle mass (kg)	34.8	2.3	29.7	2.9	0.211	0.58
Muscle mass (%)	33.8	1.2	32.3	2.1	0.590	0.25
*Fat mass*						
Fat mass (kg)	34.2	2.1	28.9	4.6	0.994	−0.00
Fat mass (%)	28.0	2.5	29.6	3.1	0.365	−0.42
*Anthropometric measures*						
Waist circumference (cm)	105.2	5.3	104.9	2.0	0.472	0.33
Hip circumference (cm)	108.3	4.7	107.2	2.2	0.121	−0.73
*Metabolic health*						
Visceral fat (%)	14.2	1.3	11.8	1.3	0.259 ^a^	0.52

Note: ^a^ Mann–Whitney U test was used due to violation of normality (Shapiro–Wilk *p* = 0.007).

**Table 3:** Pre- and post-intervention values for body composition and anthropometric measures.

	S1RM Group (n = 10)			App Group (n = 10)		
Variable	Pre	Post	Δ	Pre	Post	Δ
Weight (kg)	98.4 ± 4.5	92.1 ± 3.8	−6.3	97.6 ± 7.7	94.5 ± 7.2	−3.1
BMI (kg/m^2^)	31.6 ± 0.8	29.4 ± 0.7	−2.2	32.1 ± 1.6	31.1 ± 1.5	−1.0
Muscle mass (kg)	34.8 ± 2.3	36.2 ± 2.1	+1.4	29.7 ± 2.9	30.9 ± 3.1	+1.2
Muscle mass (%)	33.8 ± 1.2	35.2 ± 0.9	+1.4	32.3 ± 2.1	33.6 ± 1.8	+1.3
Fat mass (kg)	34.2 ± 2.1	30.8 ± 1.9	−3.4	28.9 ± 4.6	28.3 ± 2.9	−0.6
Fat mass (%)	28.0 ± 2.5	26.0 ± 1.8	−2.0	29.6 ± 3.1	28.5 ± 2.6	−1.1
Waist (cm)	105.2 ± 5.3	98.7 ± 4.6	−6.5	104.9 ± 2.0	103.9 ± 1.9	−1.0
Hip (cm)	108.3 ± 4.7	102.5 ± 3.9	−5.8	107.2 ± 2.2	107.5 ± 2.2	+0.3
Visceral fat (%)	14.2 ± 1.3	13.6 ± 1.2	−0.6	11.8 ± 1.3	11.3 ± 0.8	−0.5

Note: Values are presented as Mean ± SD. ∆ = Mean difference (Post – Pre).

**Table 4:** Mixed repeated-measures ANOVA results (Group × Time) with effect sizes.

	Time		Group		Time × Group	
Variable	F	*p* ($\eta^{2}p$)	F	*p* ($\eta^{2}p$)	F	*p* ($\eta^{2}p$)
Weight (kg)	189.10	<0.001 (0.91)	2.11	0.164 (0.11)	0.19	0.671 (0.01)
BMI (kg/m^2^)	232.12	<0.001 (0.93)	0.98	0.336 (0.05)	0.45	0.511 (0.02)
Muscle mass (kg)	46.65	<0.001 (0.72)	2.22	0.154 (0.11)	4.13	0.057 (0.19)
Muscle mass (%)	271.41	<0.001 (0.94)	0.50	0.489 (0.03)	3.16	0.093 (0.15)
Fat mass (kg)	6.43	0.021 (0.26)	2.34	0.144 (0.12)	3.43	0.080 (0.19)
Fat mass (%)	1.92	0.182 (0.10)	0.13	0.723 (0.01)	3.08	0.096 (0.18)
Waist (cm)	44.80	<0.001 (0.71)	4.41	0.050 (0.20)	**14.50**	**0.001 (0.45)**
Hip (cm)	92.00	<0.001 (0.84)	0.32	0.579 (0.02)	**217.90**	**<0.001 (0.92)**
Visceral fat (%)	11.77	0.003 (0.40)	**4.91**	**0.040 (0.21)**	2.00	0.175 (0.10)

Note: $\eta^{2}p$ = partial eta squared. Bold values indicate statistical significance (*p* < 0.05). Large effect sizes ($\eta^{2}p$ ≥ 0.14) are observed for most Time effects and for Time × Group interactions in waist, hip, muscle mass, and fat mass variables.

**Table 5:** Pre–post differences with 95% confidence intervals.

	S1RM Group (n = 10)		App Group (n = 10)	
Variable	Δ (Post − Pre)	95% CI	Δ (Post − Pre)	95% CI
Weight (kg)	−6.30	[−8.15, −4.45]	−3.48	[−4.85, −2.11]
BMI (kg/m^2^)	−2.20	[−2.85, −1.55]	−1.00	[−1.48, −0.52]
Muscle mass (kg)	+1.40	[+0.52, +2.28]	+1.20	[+0.45, +1.95]
Muscle mass (%)	+1.40	[+1.05, +1.75]	+1.30	[+0.89, +1.71]
Fat mass (kg)	−3.40	[−5.62, −1.18]	−0.60	[−2.18, +0.98]
Fat mass (%)	−2.00	[−3.42, −0.58]	−1.10	[−2.35, +0.15]
Waist (cm)	−6.50	[−9.82, −3.18]	−1.00	[−1.52, −0.48]
Hip (cm)	−5.80	[−8.15, −3.45]	+0.30	[−0.48, +1.08]
Visceral fat (%)	−0.60	[−1.42, +0.22]	−0.50	[−1.08, +0.08]

Note: Δ = Mean difference (Post − Pre). CI = Confidence interval. Negative values indicate reduction; positive values indicate increase.

**Table 6:** Changes in estimated maximal strength (indirect 1RM) in the S1RM group (n = 10).

Exercise	Pre (kg)	Post (kg)	Increase (%)	*p*-Value
Chest press	65.3 ± 4.1	77.2 ± 3.8	18.2	<0.001
Pull down	72.8 ± 5.2	84.1 ± 4.7	15.5	0.001
Shoulder press	40.5 ± 3.6	48.6 ± 3.2	20.0	<0.001
Triceps press	35.7 ± 2.8	41.5 ± 2.3	16.2	<0.001
Biceps curl	28.4 ± 2.5	32.8 ± 2.1	15.5	<0.001
Leg extension	50.2 ± 3.8	61.2 ± 3.5	22.0	<0.001
Seated leg curl	45.6 ± 3.9	52.4 ± 3.2	14.9	0.009

## Data Availability

The data will be made available upon request to the corresponding author. The data are not publicly available due to containing information that could compromise the privacy of research participants.
